# HPV vaccination associates with *Lactobacillus crispatus*-dominant vaginal microbiota in women with cervical lesions: a metagenomic study

**DOI:** 10.3389/fmicb.2026.1906211

**Published:** 2026-08-14

**Authors:** Ruvie Pearl Araneta, Yeseul Choi, Jung Hwa Lee, Heon Jong Yoo, Yoo-Young Lee, Angela Cho, Hee Yeun Yoon, Jong Mi Kim, Nora Jee-Young Park, Incheol Seo, Gun Oh Chong, Chul Min Park

**Affiliations:** 1 Department of Immunology, School of Medicine, Kyungpook National University, Daegu, Republic of Korea; 2 Clinical Omics Institute, Kyungpook National University, Daegu, Republic of Korea; 3 Center for Gynecologic Cancer, National Cancer Center, Goyang, Republic of Korea; 4 Chungnam National University School of Medicine, Daejeon, Republic of Korea; 5 Department of Obstetrics and Gynecology, Gynecologic Cancer Center, Samsung Medical Center, Sungkyunkwan University School of Medicine, Seoul, Republic of Korea; 6 Department of Obstetrics and Gynecology, Jeju National University Hospital, Jeju National University College of Medicine, Jeju, Republic of Korea; 7 Department of Obstetrics and Gynecology, Kyungpook National University Chilgok Hospital, Daegu, Republic of Korea; 8 Department of Obstetrics and Gynecology, Kyungpook National University, Daegu, Republic of Korea; 9 Department of Pathology, Kyungpook National University Chilgok Hospital, Daegu, Republic of Korea

**Keywords:** cervical lesions, high-risk HPV, HPV vaccination, human papillomavirus, shotgun metagenomics, vaginal microbiota

## Abstract

**Background:**

Human papillomavirus (HPV) vaccination provides high-level protection against cervical cancer through type-specific antibodies, although breakthrough and persistent high-risk HPV (hrHPV) infections may still occur in some vaccinated women. In addition to neutralizing antibodies, vaccination elicits other immune mechanisms that may influence viral persistence and disease progression. Because mucosal immunity is closely linked to the vaginal microbiota, microbial composition may also play an important role in HPV susceptibility, with *Lactobacillus crispatus* dominance associated with protection, and dysbiosis with impaired mucosal immunity. Whether HPV vaccination is associated with favorable microbiota signatures in women with confirmed cervical cancer-related conditions remains unknown.

**Method:**

We analyzed vaginal samples via shotgun metagenomic sequencing from a cross-sectional study of 59 pre-menopausal women (<50 years) with confirmed cervical lesions (HPV-unvaccinated *n* = 26, vaccinated *n* = 33).

**Results:**

Alpha diversity (Shannon and Inverse Simpson indices) did not differ significantly by vaccination status, suggesting within-sample microbial diversity between the groups, but Bray-Curtis-based PERMANOVA indicated a statistically significant difference in overall community composition between groups (*p* = 0.042). Among women with CST I, 90.9% were vaccinated, whereas among women with CST IV, 60.0% were unvaccinated. At the species level, *L. crispatus* relative abundance was higher in vaccinated women in the unadjusted analysis; however, this association was attenuated and was not statistically significant after multivariable adjustment. HPV vaccination status was associated with a higher prevalence of *L. crispatus*-dominant, health-associated vaginal microbiota among women with cervical lesions.

**Conclusion:**

These findings support the hypothesis that HPV vaccination status is associated with *L. crispatus* dominance in this population, but mechanistic pathways remain to be elucidated.

## 1 Introduction

Cervical cancer (CC) remains a major cause of disease and fatality among women. In 2022, an estimated 662,044 new CC cases globally, increasing by 1%−2% annually, with an overwhelming majority of deaths occurring in countries with lowest Human Development Index (HDI), where access to screening and vaccination remains limited (Das, 2021; Siegel et al., 2024; Wu J. et al., 2025). High-risk human papillomavirus (hrHPV) persistent infection, particularly types 16 and 18, is firmly established as a necessary etiologic factor, causing 71%−76% of CC, based on decades of molecular, epidemiological, and natural history studies (de Sanjose et al., 2010; World Health Organization, 2025).

Prophylactic HPV vaccines have transformed the landscape of CC prevention. Current licensed vaccines—bivalent, quadrivalent, and nonavalent vaccines—induce robust neutralizing antibody responses against the L1 capsid proteins of oncogenic HPV types (Seong and Ryou, 2020; Roy et al., 2023). Clinical trials and long-term follow-up cohorts consistently demonstrate 90%−95% efficacy in preventing persistent vaccine-type HPV infection and high-grade cervical intraepithelial neoplasia (CIN) among HPV-naïve individuals at vaccination, with near-complete protection against HPV16/18-related high-grade lesions. Work synthesized by Stanley and colleagues delineated the mechanistic basis of this protection as high-titer, type-specific neutralizing antibodies that prevent virion binding and entry at the cervical transformation zone (Stanley, 2007). More recent studies have further shown that current vaccines induce high seroconversion rates and antibody titers that substantially exceed those after natural infection, and protection against vaccine-type HPV infection and related high-grade cervical lesions has been maintained for up to around a decade in long-term follow-up, with no clear evidence of waning efficacy to date (Arbyn et al., 2018; Roy et al., 2023).

However, accumulating evidence indicates that HPV vaccination, while highly effective, is not absolute (Reuschenbach et al., 2023). A proportion of vaccinated women—a small minority, with risk strongly influenced by age at vaccination, sexual exposure history, and behavioral and clinical risk factors—still develop persistent HPV infection or HPV-related cervical disease (Herweijer et al., 2016). Long-term cohort data from Nordic populations and recent analyses in adolescent and young adult cohorts have documented breakthrough infections and even high-grade lesions among fully vaccinated women, albeit at substantially reduced rates compared with unvaccinated peers (Leval et al., 2013; Lehtinen et al., 2017; Dehlendorff et al., 2021; Kjaer et al., 2021; Wu S. et al., 2025). These breakthrough events, together with mechanistic data from Roy et al., raise the possibility that non-neutralizing antibody functions—such as Fc-mediated effector functions [antibody-dependent cellular phagocytosis (ADCP), antibody-dependent cellular cytotoxicity (ADCC)], and vaccine-induced cellular immunity—may contribute importantly to determining which vaccinated individuals clear or progress after exposure, beyond what neutralizing titers alone would predict (Roy et al., 2023).

Parallel advances in vaginal microbiota (VM) research have revealed that the cervicovaginal microbial ecosystem is an important determinant of susceptibility to HPV acquisition, persistence, and progression to CIN (Jung et al., 2025; Leon-Gomez and Romero, 2025). The community state type (CST) framework introduced by Ravel and colleagues and refined in subsequent work, including updated classifications by France et al. has provided a structured way to characterize vaginal communities (Ravel et al., 2011). In reproductive-age women, “healthy” microbiota is typically dominated by one of several *Lactobacillus* species, giving rise to distinct CSTs (e.g., CST I, *L. crispatus*; CST II, *L. gasseri*; CST III, *L. iners*; CST V, *L. jensenii*), whereas CST IV encompasses diverse, *Lactobacillus*-depleted, anaerobe-rich communities. A consistent finding across studies is that *L. crispatus*–dominant CST I is associated with the most favorable gynecologic outcomes, including lower rates of bacterial vaginosis (BV), sexually transmitted infections, and hrHPV persistence, whereas CST IV dysbiosis—characterized by *Gardnerella, Prevotella, Atopobium, Sneathia*, and other anaerobes—is strongly linked to increased HPV acquisition, persistence, and progression to high-grade lesions (Ravel et al., 2011; France et al., 2020; Norenhag et al., 2020; Wei et al., 2022; Yang et al., 2025). Recent cohort and mechanistic work further reinforces the concept that vaginal dysbiosis may contribute causally to HPV-related disease risk (Cheng et al., 2020; Zhai et al., 2021; Liu Y. et al., 2023). To our knowledge, there are currently no or very limited data on cervicovaginal microbiota stratified by HPV vaccination status in women with cervical lesions.

Mechanistically, the VM interfaces intimately with mucosal immunity (Castanheira et al., 2021; Nicolò et al., 2021; Dong et al., 2023). *Lactobacillus* species, particularly *L. crispatus*, creates an environment hostile to many pathogens and favoring epithelial homeostasis by maintaining a low vaginal pH through lactic acid production. Lactic acid, which exists as L- and D-isomers, modulates epithelial barrier function, tight junction integrity, and local cytokine milieu, and can directly influence the activity of resident and recruited immune cells (Nicolò et al., 2021; Schwecht et al., 2023). Lactobacilli also produce hydrogen peroxide (H_2_O_2_) and bacteriocins that inhibit overgrowth of dysbiotic and potentially pathogenic taxa, and they support mucosal IgA responses and antimicrobial peptide expression. In contrast, dysbiotic communities enriched for *Gardnerella, Prevotella*, and related anaerobes secrete proteases, sialidases, and other virulence factors that degrade cervical mucus and epithelial junctions, increase permeability, and drive chronic low-grade inflammation. This inflammatory microenvironment—with elevated IL-6, IL-8, TNF-α and shifts in Th17 and regulatory T-cell responses—is associated with impaired local antiviral immunity and a higher likelihood of HPV persistence (Nicolò et al., 2021; Dong et al., 2023; Sheng et al., 2025). In this way, the VM acts as both a structural and immunological gatekeeper shaping the outcome of HPV exposure and potentially influencing the effectiveness of naturally acquired and vaccine-induced immune responses (Nicolò et al., 2021; Bautista et al., 2025).

Taken together, these lines of evidence suggest a tripartite interaction among HPV, the host immune system, and the VM. Yet, a critical question remains unanswered: does HPV vaccination status associate with a more protective VM profile, particularly in women who already have cervical disease? Most vaccine trials and effectiveness studies have focused on clinical endpoints (HPV infection, CIN, CC) and serological readouts, without systematically characterizing the cervicovaginal microbiome. Conversely, microbiome studies in HPV-infected and CIN/CC populations have seldom incorporated detailed vaccination histories or immunologic profiling. It thus remains unclear whether vaccinated women who nonetheless develop HPV-related cervical disease exhibit microbiota that are more, less, or equally dysbiotic compared with unvaccinated women with similar lesions. This uncertainty prompts the hypothesis that vaccination-associated immune shifts could, in principle, be reflected in the structure or function of the VM, although only small exploratory studies in healthy women have begun to address this, and no systematic data exist in women with cervical disease (Giraldo et al., 2021; Bautista et al., 2025).

This research gap has important implications. If HPV vaccination, even in the setting of breakthrough disease, is associated with greater *L. crispatus* dominance or reduced dysbiosis in women with cervical disease, this could raise the possibility that vaccine-induced immune responses may be linked to a more favorable vaginal microbial environment. Such an association may provide a basis for future studies examining whether vaccination and VM jointly influence HPV clearance, lesion progression, or treatment response.

In this context, the present study investigates the association between HPV vaccination status and the composition of the VM in women with cervical lesion–related disease (pre-invasive and invasive). Specifically, it tests the hypothesis that HPV vaccination status is associated with a higher prevalence of protective *Lactobacillus* spp. and reduced dysbiosis among women with confirmed cervical lesions. Any potential involvement of vaccination-related changes in mucosal immunity remains to be investigated. By integrating detailed vaccination histories, HPV genotype and persistence data, and high-resolution vaginal microbiome profiling, this work aims to identify VM patterns associated with HPV vaccination status and to generate hypotheses regarding the potential relationships among vaccination, mucosal immunity, and the VM. Ultimately, these associations may provide a basis for future investigation of microbiota-targeted adjunctive strategies and risk stratification, pending prospective validation.

While the associations between *L. crispatus* dominance and favorable HPV outcomes, and between CST IV dysbiosis and HPV persistence, are well established in the literature, these relationships have not previously been examined through the lens of vaccination status in a population with confirmed cervical lesions. The novelty of this study lies in demonstrating, using high-resolution shotgun metagenomics, that HPV vaccination status—rather than HPV infection status alone—is associated with distinct patterns of vaginal community composition in women with cervical lesions, even after disease has already developed.

## 2 Materials and methods

### 2.1 Study population

This study was approved by the Institutional Review Board of Kyungpook National University Chilgok Hospital (KNUCH 2023-08-054). Participants were pre-menopausal women (< 50 years) attending the Department of Obstetrics and Gynecology at Kyungpook National University Chilgok Hospital (Daegu, Republic of Korea) with histologically confirmed cervical lesions. From 98 recruited women, 39 were excluded due to menopause or age ≥50 years, leaving 59 eligible participants for analysis. HPV vaccination status and available details were ascertained from medical records.

### 2.2 Vaginal swab samples collection and HPV genotyping

The swab samples were obtained from the 59 women. Gynecologists performed the sample collection of cervicovaginal specimens. Participants were placed in the lithotomy position, and a speculum lubricated with sterile saline solution was inserted by gynecologists. Vaginal swabs (CORE-SWAB, Incore, Republic of Korea) were rotated gently for 5 s against the midvaginal wall—at the midpoint between the vaginal introitus and the posterior fornix—to collect secretions while minimizing vaginal mucosa irritation (Seo et al., 2026). After collection, swabs were snapped at the marked cutting line and immediately transferred to DNase, RNase, and DNA-free 15 ml tubes (SPL Life Science, Pocheon, Republic of Korea). Immediately after collection, samples were delivered to the laboratory within 3 days, then stored at 4 °C until DNA extraction, which was performed within 3 days of receipt. HPV genotyping was performed using Anyplex II HPV28 Detection Kit (Seegene, Seoul, Republic of Korea) per the manufacturer's instructions, as previously detailed by Jung and colleagues (Jung et al., 2025).

Vaccination-related information, including vaccine type, age at vaccination, and time since vaccination, was collected through participant questionnaires and supplemented or verified using available medical records. Vaccine coverage of detected HPV genotypes was determined according to the administered HPV vaccine formulation.

### 2.3 Microbial DNA extraction and shotgun metagenomic sequencing

Total microbial DNA was isolated using the DNeasy^®^ PowerSoil^®^ Pro DNA Kit (QIAGEN, Hilden, Germany) following the manufacturer's protocol. Following DNA extraction, host DNA was depleted using the NEBNext^®^ Microbiome DNA Enrichment Kit (New England Biolabs, Ipswich, Massachusetts, USA) according to the manufacturer's protocol. DNA concentration and purity were assessed via NanoDrop™ 2000c (Thermo Fisher, Waltham, Massachusetts, USA). Sequencing libraries were constructed at Macrogen (Seoul, Republic of Korea) using the TruSeq Nano DNA High Throughput Library Prep Kit (Illumina, San Diego, California, USA) per the manufacturer's protocol. In brief, 100 ng genomic DNA underwent acoustic shearing (Covaris, Woburn, Massachusetts, USA) followed by end-repair to yield 5′-phosphorylated blunt-ended fragments, then bead-based size selection. Indexed adapters (TruSeq DNA UD) were subsequently ligated after “A” base addition, with PCR-based enrichment and purification to complete library preparation. Libraries were quantitated via qPCR (KAPA Library Quantification Kit, following Illumina guidelines) and sized on an Agilent 4200 TapeStation using D1000 ScreenTape (Agilent technologies, Santa Clara, California, USA). Paired-end sequencing was performed on the Illumina NovaSeq platform (Seo et al., 2026).

### 2.4 Bioinformatic analysis

Shotgun metagenomic reads were taxonomically profiled using the *nf-core/taxprofiler* pipeline (version 1.1.8) implemented in *Nextflow* (version 24.10.5). Raw sequencing reads were first subjected to quality control using *Falco*. Host-derived reads were removed by mapping reads against the human reference genome GRCh38.p14 (GCF_000001405.40) using *Bowtie2*, and only non-host reads were retained for downstream analysis. Taxonomic profiling was performed using MetaPhlAn (version 4.0.6) with the mpa_vJun23_CHOCOPhlAnSGB_202403 database. The resulting taxonomic abundance tables were used for downstream microbial community analyses. All downstream analyses were performed in R (version 4.5.0) using the *phyloseq, vegan, cluster*, and *ggplot2* packages.

Alpha diversity was calculated using Shannon and Inverse Simpson indices at genus and species levels. Beta diversity was assessed using Bray-Curtis dissimilarity on species-level relative abundances and visualized by principal coordinates analysis (PCoA). Microbial community structure was further explored using *k-means* clustering based on the first two PCoA axes, with the optimal number of clusters determined using the elbow criterion.

CSTs were assigned according to the dominant *Lactobacillus* species. Samples dominated by *L. crispatus, L. gasseri, L. iners*, or *L. jensenii* were classified as CST I, II, III, and V, respectively. Samples without a dominant species at a relative abundance of ≥0.5 were classified as CST IV.

### 2.5 Statistical analysis

For demographic comparisons according to HPV vaccination status, continuous variables were assessed for normality using Shapiro–Wilk test and compared using Wilcoxon rank-sum test or Kruskal–Wallis test, as appropriate. Categorical variables were compared using the chi-square or Fisher's exact test.

At the community level, associations between HPV vaccination status and overall VM composition were evaluated using Permutational Multivariate Analysis of Variance (PERMANOVA) (adonis2 function, *vegan*) with 999 permutations on Bray–Curtis dissimilarities derived from species-level relative abundances. To assess whether the significant PERMANOVA results could be driven by differences in within-group dispersion, we additionally tested homogeneity of multivariate dispersions using Permutational Multivariate Analysis of Dispersion (PERMDISP) (betadisper function, *vegan*).

For cluster- and CST-level analyses, differences in demographic and clinical variables including vaccination status, across microbiome-defined clusters and CSTs were assessed using Kruskal–Wallis tests for continuous variables and chi-square or Fisher's exact tests for categorical variables, as appropriate.

For species-level analyses of four key taxa (*L. crispatus, L. iners, G. vaginalis*, and *B. breve*), relative abundances were compared between unvaccinated and vaccinated women using Wilcoxon rank-sum tests, and *p-*values from these tests were displayed on the corresponding boxplots.

To evaluate the association between HPV vaccination and *L. crispatus* relative abundance after adjustment of potential confounders, multivariable linear regression analysis was performed. *L. crispatus* relative abundance was arcsine square-root transformed before the analysis. HPV vaccination status was included as the primary exposure, and age, hormone use, antibiotics, probiotics, disease severity, HPV type (HPV-16/18 vs. other), and multiple HPV infection were included as covariates. A sensitivity analysis excluding antibiotics and disease severity was additionally performed due to sparse observations in these categories. Regression coefficients, 95% confidence intervals, and two-sided *p-*values were reported.

## 3 Results

### 3.1 Study cohort characteristics

Of the 98 women with histologically confirmed cervical lesions initially enrolled, 39 post-menopausal or ≥50 years of age were excluded according to the pre-defined criteria, leaving 59 pre-menopausal (< 50 years) for analysis (Supplementary Figure 1). Among the 59 participants, 26 were unvaccinated and 33 reported prior HPV vaccination. Baseline demographic and clinical characteristics by vaccination status are summarized in Table 1. Unvaccinated women (*n* = 26; mean age 39.08 ± 5.74 years, body weight 59.03 ± 13.63 kg) and vaccinated women (*n* = 33; mean age 36.3 ± 6.1 years, body weight 61.06 ± 16.95 kg) showed comparable baseline demographics. No statistically significant differences were detected in the measured baseline characteristics, although vaccinated women were numerically younger and more frequently college educated than unvaccinated women. Disease severity (pre-invasive lesions vs. CC) was similar between groups (22/26 vs. 31/33 pre-invasive; *p* > 0.05). Similarly, there were no statistically significant differences between groups with respect to the use of antibiotics, hormones, and probiotics, smoking and drinking status, or disease condition (all *p* > 0.05 by Wilcoxon rank-sum test for continuous variables and chi-square, or Fisher's exact test for categorical variables, as appropriate), indicating broadly comparable groups at baseline.

**Table 1:** Baseline characteristics of study participants by HPV vaccination status (*n* = 59).

Characteristics	Unvaccinated (*n* = 26)	Vaccinated (*n* = 33)	*p*-value
Age (mean ± SD)	39.08 ± 5.74	36.3 ± 6.1	0.207
Body weight, kg (mean ± SD)	59.03 ± 13.63	61.06 ± 16.95	0.414
Educational attainment (*n*, %)			0.350
High school graduate	11 (42.3)	9 (27.3)	
College graduate	15 (57.7)	24 (72.7)	
Disease severity			0.390
Pre-invasive	22 (84.6)	31 (93.9)	
Cervical Cancer	4 (15.4)	2 (6.1)	
HPV infection (*n*, %)			1.000
No	0 (0.0)	1 (3.0)	
Yes	26 (100.0)	32 (97.0)	
HPV infection category (*n*, %)			0.885
No	0 (0.0)	1 (3.0)	
Single	15 (57.7)	17 (51.5)	
Multiple	11 (42.3)	15 (45.5)	
HPV type (*n*, %)			0.386
Negative	0 (0.0)	1 (3.0)	
Low risk	0 (0.0)	2 (6.1)	
16/18	13 (50.0)	11 (33.3)	
Other high risk	13 (50.0)	19 (57.6)	
Antibiotics (*n*, %)			0.578
No	24 (92.3)	32 (97.0)	
Yes	2 (7.7)	1 (3.0)	
Hormones (*n*, %)			0.718
No	23 (88.5)	27 (81.8)	
Yes	3 (11.5)	6 (18.2)	
Probiotics (*n*, %)			0.566
No	9 (34.6)	15 (45.5)	
Yes	17 (65.4)	18 (54.5)	
Smoking (*n*, %)			0.713
No	19 (73.1)	22 (66.7)	
Yes	4 (15.4)	4 (12.1)	
Ex-smoker	3 (11.5)	7 (21.2)	
Drinking (*n*, %)			0.893
No	10 (38.5)	11 (33.3)	
Yes	16 (61.5)	22 (66.7)	
Diabetes (*n*, %)			1.000
No	26 (100.0)	32 (97.0)	
Yes	0 (0.0)	1 (3.0)	
Hypertension (*n*, %)			0.248
No	26 (100.0)	30 (90.9)	
Yes	0 (0.0)	3 (9.1)	

Study participants were women with pathologically confirmed cervical lesions visiting the gynecology clinic, Kyungpook National University Chilgok Hospital.

HPV vaccination status was ascertained from medical records. Continuous variables were assessed for normality using the Shapiro–Wilk test and compared between HPV-vaccinated and unvaccinated groups using the Wilcoxon rank-sum test.

Categorical variables were compared using Fisher's exact test (expected cell count < 5) or the chi-square test (expected cell count ≥5), as appropriate.

In this cervical lesion cohort, hrHPV infection was highly prevalent. In unvaccinated women, 26/26 (100.0%) were hrHPV-positive, while among vaccinated women 30/33 (90.9%) were hrHPV-positive, 2/33 (6.1%) were low-risk HPV positive, and 1/33 (3.0%) was HPV-negative. Overall, 58/59 women had detectable HPV infection (hrHPV or low-risk HPV). The distribution of hrHPV genotypes was also similar by vaccination status. No significant differences in overall HPV positivity, hrHPV types, or multiple-type of infection were observed between unvaccinated and vaccinated women (all *p* > 0.05 by chi-square or Fisher's exact test, as appropriate).

Among the 33 vaccinated women, 14 received the quadrivalent HPV vaccine (Gardasil4) and 17 received the nonvalent HPV vaccine (Gardasil9), while specific vaccine type was unavailable for two patients. Among the 32 patients with available vaccination records, the mean age at vaccination was 29.3 ± 6.8 years, and the mean interval between the last vaccination and sample collection was 6.2 ± 5.5 years. According to the administered vaccine formulation, both vaccine-covered and non-vaccine covered HPV genotypes were detected among recipients of the quadrivalent and nonavalent vaccines (Table 2).

**Table 2:** Distribution of HPV genotypes detected among women receiving HPV vaccination by vaccine type and coverage.

HPV genotype	Gardasil4		Gardasil9	
	Coverage category	*n* (%)	Coverage category	*n* (%)
HPV-6	Covered	0 (0.0%)	Covered	1 (5.9%)
HPV-11	Covered	0 (0.0%)	Covered	0 (0.0%)
HPV-16	Covered	4 (28.6%)	Covered	6 (35.3%)
HPV-18	Covered	0 (0.0%)	Covered	1 (5.9%)
HPV-31	Non-covered	2 (14.3%)	Covered	2 (11.8%)
HPV-33	—	—	Covered	3 (17.6%)
HPV-35	—	—	Non-covered	1 (5.9%)
HPV-39	Non-covered	1 (7.1%)	—	—
HPV-45	—	—	Covered	0 (0.0%)
HPV-51	Non-covered	4 (28.6%)	—	—
HPV-52	Non-covered	3 (21.4%)	Covered	0 (0.0%)
HPV-53	Non-covered	1 (7.1%)	Non-covered	2 (11.8%)
HPV-56	Non-covered	2 (14.3%)	Non-covered	2 (11.8%)
HPV-58	Non-covered	3 (21.4%)	Covered	1 (5.9%)
HPV-68	Non-covered	1 (7.1%)	—	—
HPV-70	Non-covered	2 (14.3%)	Non-covered	3 (17.6%)
Low-risk HPV (genotype unspecified)	Not classifiable	2 (14.3%)	Not classifiable	3 (17.6%)

For each HPV genotype, detection frequencies *n* (%) are shown separately for Gardasil4 and Gardasil9 recipients and classified as vaccine-covered, non-vaccine-covered, or not classifiable according to the administered vaccine formulation.

### 3.2 Microbial community structure by vaccination status

**Figure 1:**
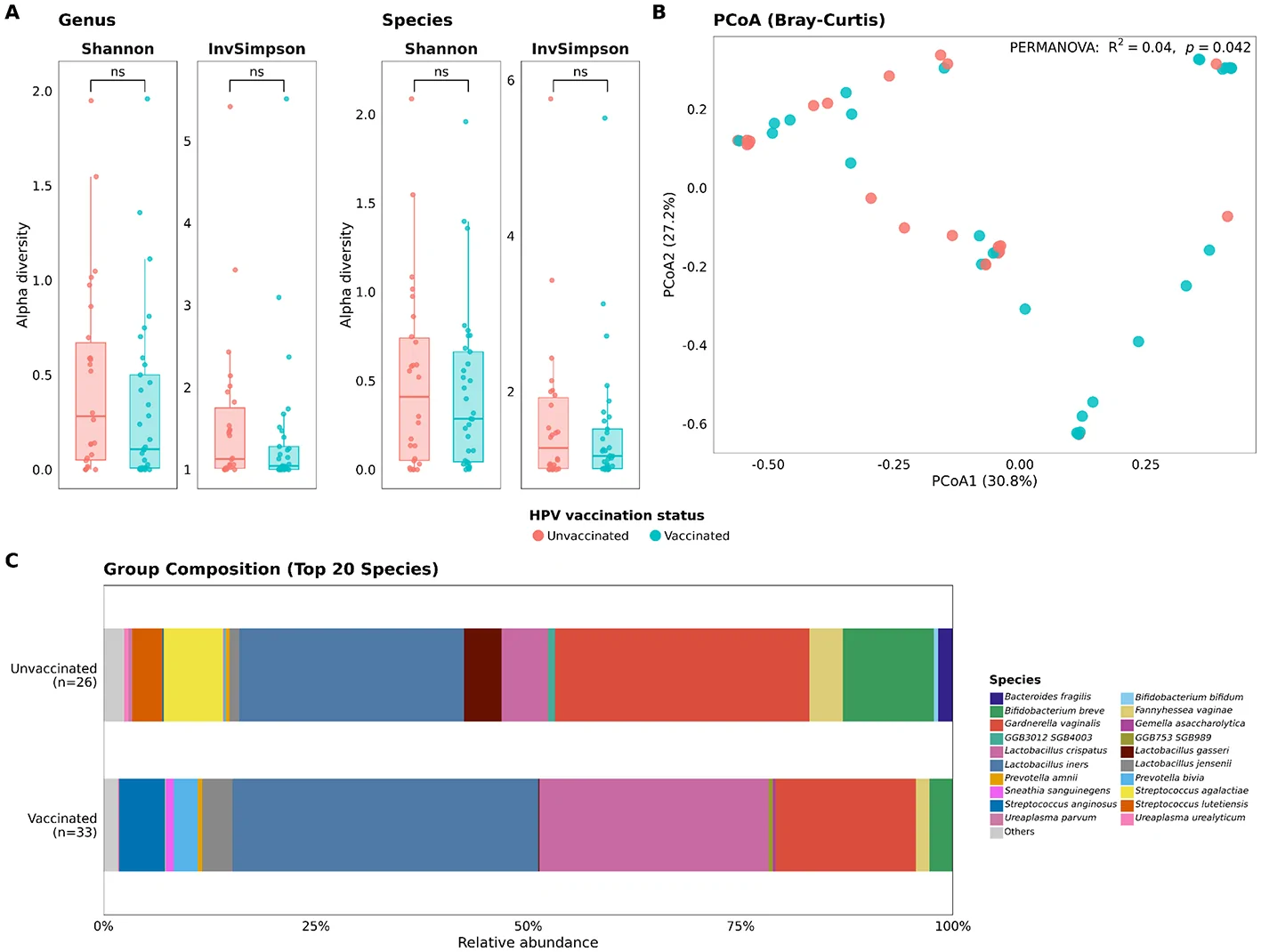
Community-level vaginal microbiota by HPV vaccination status. **(A)** Alpha diversity of vaginal microbiota in HPV-unvaccinated (*n* = 26) and vaccinated (*n* = 33) women, assessed using Shannon and Inverse Simpson indices at both genus and species levels based on MetaPhlAn-derived relative abundances. Horizontal lines indicate medians; boxes the interquartile range (IQR); whiskers, 1.5 x IQR. “ns” denotes non-significant differences (*p* > 0.05). **(B)** PCoA of Bray–Curtis dissimilarities derived from species-level relative abundances. PERMANOVA was used to test for differences in overall community composition between vaccination groups. To assess whether this result could be driven by unequal dispersion, PERMDISP was performed. **(C)** Stacked bar plots showing the relative abundance of the top 20 bacterial species in HPV-unvaccinated and vaccinated women. Bars represent individual samples grouped by vaccination status, with colors indicating species.

We first assessed the alpha diversity of VM by HPV vaccination status using Shannon and Inverse Simpson indices at both genus and species levels, which capture within-sample richness and evenness of the community. Across taxonomic levels, both indices showed no significant differences between unvaccinated and vaccinated samples, suggesting comparable overall richness and evenness (Figure 1A).

In contrast, PCoA of Bray–Curtis dissimilarities revealed a significant difference in overall community composition by vaccination status based on PERMANOVA (*R*^2^ = 0.04, *p* = 0.042; Figure 1B). Because PERMANOVA results can be influenced by differences in within-group dispersion, we evaluated multivariate dispersions using PERMDISP, which showed no significant difference between unvaccinated and vaccinated groups (*p* = 0.783), suggesting that the observed compositional difference was not driven by heterogeneity of dispersion. Taxonomic profiles by vaccination are shown in stacked bar plots of the top 20 species, where *L. iners* and *L. crispatus* exhibited higher relative proportions in vaccinated women than in unvaccinated women (Figure 1C).

### 3.3 Cluster-level community types and CSTs

**Figure 2:**
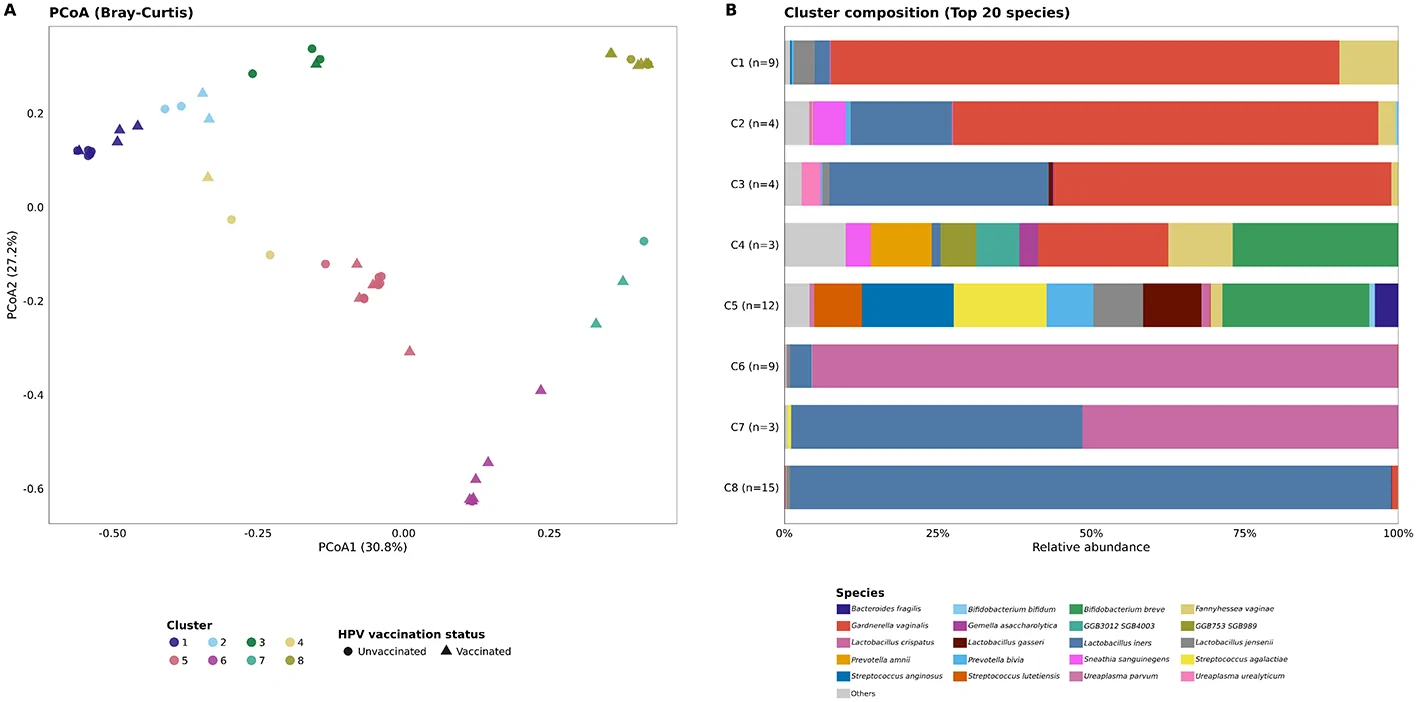
Vaginal microbiota clusters and taxonomic composition (*k* = 8). **(A)** PCoA ordination of Bray–Curtis dissimilarities at the species level for 59 vaginal samples, colored by eight microbiota clusters (C1–C8) obtained by k-means clustering on the first two PCoA axes. The number of clusters (*k* = 8) was selected using an elbow-based criterion (see Supplementary Figure 2). Point shapes denote HPV vaccination status. **(B)** Stacked bar plots showing the relative abundance of the top 20 bacterial species across the eight clusters. Samples are ordered by cluster membership, and colors indicate species. Clusters exhibit distinct dominant taxa, including *G. vaginalis*-dominant clusters (1–3), *B. breve*-enriched clusters (4, 5), and *Lactobacillus*-dominant clusters (6–8).

Clusters were obtained by k-means clustering (*k* = 8) on the first two PCoA axes, consistent with the bioinformatic flow described in the Methods, and supported by elbow-plot analysis at genus and species levels (Supplementary Figure 2). PCoA of Bray–Curtis dissimilarities at the species level thus revealed eight distinct microbiome clusters (C1–8), analogous to previously reported CST ordinations (Figure 2A).

Cluster-specific taxonomic profiles are shown in the stacked bar plots of the top 20 species (Figure 2B). Clusters C1–C5 were characterized by reduced *Lactobacillus* and enrichment of diverse, anaerobe-rich communities including *G. vaginalis* (C1–C3), consistent with dysbiotic CST IV. Clusters C4–C5 showed similar diversity patterns with notable *B. breve* enrichment, while clusters C6–C8 exhibited high relative abundance of *L. crispatus* and *L. iners*, corresponding to CST I and CST III community types.

Participants characteristics by microbiome-defined cluster are summarized in Table 3. Age and body weight did not differ significantly across clusters (all *p* > 0.05), suggesting that these variables were not major contributors to cluster separation in this cohort. Similarly, overall HPV infection and hrHPV status was comparably distributed across clusters. Vaccination prevalence varied numerically across clusters but did not reach statistical significance at the cluster level (*p* = 0.257). Disease severity (pre-invasive lesions vs. CC) also differed across clusters (*p* = 0.038), although the small number of CC cases per cluster limits interpretation of specific patterns.

**Table 3:** Baseline characteristics of study participants by vaginal microbiota community cluster.

Variables	Cluster 1 (*n* = 9)	Cluster 2 (*n* = 4)	Cluster 3 (*n* = 4)	Cluster 4 (*n* = 3)	Cluster 5 (*n* = 12)	Cluster 6 (*n* = 9)	Cluster 7 (*n* = 3)	Cluster 8 (*n* = 15)	*p*-value
Age (mean ± SD)	37.67 ± 4.06	37 ± 10.42	35.75 ± 4.19	42.67 ± 7.77	40.25 ± 6.18	34.22 ± 7.31	39.33 ± 9.61	36.47 ± 3.31	0.249
Body weight, kg (mean ± SD)	55.83 ± 5.13	54.65 ± 8.68	53.98 ± 10.7	57.23 ± 6.25	71.11 ± 23.13	57.88 ± 12.17	55.53 ± 2.08	60.01 ± 16.78	0.730
Educational attainment (*n*, %)									0.262
High school graduate	2 (22.2)	3 (75.0)	1 (25.0)	1 (33.3)	7 (58.3)	2 (22.2)	1 (33.3)	3 (20.0)	
College graduate	7 (77.8)	1 (25.0)	3 (75.0)	2 (66.7)	5 (41.7)	7 (78.8)	2 (66.7)	12 (80.0)	
Disease severity (*n*, %)									0.038^*^
Pre-invasive	9 (100.0)	3 (75.0)	2 (50.0)	2 (66.7)	12 (100.0)	9 (100.0)	3 (100.0)	13 (86.7)	
Cervical cancer	0 (0.0)	1 (25.0)	2 (50.0)	1 (33.3)	0 (0.0)	0 (0.0)	0 (0.0)	2 (13.3)	
HPV infection (*n*, %)									1.000
No	0 (0.0)	0 (0.0)	0 (0.0)	0 (0.0)	0 (0.0)	0 (0.0)	0 (0.0)	1 (6.7)	
Yes	9 (100.0)	4 (100.0)	4 (100.0)	3 (100.0)	12 (100.0)	9 (100.0)	3 (100.0)	14 (93.3)	
HPV infection category (*n*, %)									0.098
No	0 (0.0)	0 (0.0)	0 (0.0)	0 (0.0)	0 (0.0)	0 (0.0)	0 (0.0)	1 (6.7)	
Single	3 (33.3)	0 (0)	3 (75.0)	1 (33.3)	10 (83.3)	4 (44.4)	2 (66.7)	9 (60.0)	
Multiple	6 (66.7)	4 (100.0)	1 (25.0)	2 (66.7)	2 (16.7)	5 (55.6)	1 (33.3)	5 (33.3)	
HPV vaccine status (*n*, %)									0.257
No	5 (55.6)	2 (50.0)	3 (75)	2 (66.7)	7 (58.3)	1 (11.1)	1 (33.3)	5 (33.3)	
Yes	4 (44.4)	2 (50.0)	1 (25.0)	1 (33.3)	5 (41.7)	8 (88.9)	2 (66.7)	10 (66.7)	
HPV type (*n*, %)									0.480
Negative	0 (0.0)	0 (0.0)	0 (0.0)	0 (0.0)	0 (0.0)	0 (0.0)	0 (0.0)	1 (6.7)	
Low risk	0 (0.0)	0 (0.0)	0 (0.0)	0 (0.0)	1 (8.3)	0 (0.0)	1 (33.3)	0 (0.0)	
16/18	3 (33.3)	2 (50.0)	3 (75.0)	2 (66.7)	4 (33.3)	2 (22.2)	0 (0.0)	8 (53.3)	
Other high risk	6 (66.7)	2 (50.0)	1 (25.0)	1 (33.3)	7 (58.3)	7 (78.8)	2 (66.7)	6 (40.0)	
Antibiotics (*n*, %)									0.192
No	9 (100.0)	3 (75.0)	3 (75.0)	3 (100.0)	12 (100.0)	9 (100.0)	3 (100.0)	14 (93.3)	
Yes	0 (0.0)	1 (25.0)	1 (25.0)	0 (0.0)	0 (0.0)	0 (0.0)	0 (0.0)	1 (6.7)	
Hormones (*n*, %)									0.069
No	9 (100.0)	3 (75.0)	4 (100.0)	3 (100.0)	12 (100.0)	7 (77.8)	3 (100.0)	9 (60.0)	
Yes	0 (0.0)	1 (25.0)	0 (0.0)	0 (0.0)	0 (0.0)	2 (22.2)	0 (0.0)	6 (40.0)	
Probiotics (*n*, %)									0.073
No	4 (44.4)	0 (0.0)	3 (75.0)	0 (0.0)	5 (41.7)	5 (55.6)	3 (100.0)	4 (26.7)	
Yes	5 (55.6)	4 (100.0)	1 (25.0)	3 (100.0)	7 (58.3)	4 (44.4)	0 (0.0)	11 (73.3)	
Smoking (*n*, %)									0.584
No	8 (88.9)	2 (50.0)	3 (75.0)	3 (100.0)	7 (58.3)	7 (77.8)	2 (66.7)	9 (60.0)	
Yes	0 (0.0)	2 (50.0)	0 (0.0)	0 (0.0)	1 (8.3)	1 (11.1)	0 (0.0)	4 (26.7)	
Ex-smoker	1 (11.1)	0 (0.0)	1 (25.5)	0 (0.0)	4 (33.3)	1 (11.1)	1 (33.3)	2 (13.3)	
Drinking (*n*, %)									0.606
No	1 (11.1)	1 (25.0)	2 (50.0)	2 (66.7)	6 (50.0)	3 (33.3)	1 (33.3)	5 (33.3)	
Yes	8 (88.9)	3 (75.0)	2 (50.0)	1 (33.3)	6 (50.0)	6 (66.7)	2 (66.7)	10 (66.7)	
Diabetes (*n*, %)									1.000
No	9 (100.0)	4 (100.0)	4 (100.0)	3 (100.0)	12 (100.0)	9 (100.0)	3 (100.0)	14 (93.3)	
Yes	0 (0.0)	0 (0.0)	0 (0.0)	0 (0.0)	0 (0.0)	0 (0.0)	0 (0.0)	1 (6.7)	
Hypertension (*n*, %)									1.000
No	9 (100.0)	4 (100.0)	4 (100.0)	3 (100.0)	11 (91.7)	8 (88.9)	3 (100.0)	14 (93.3)	
Yes	0 (0.0)	0 (0.0)	0 (0.0)	0 (0.0)	1 (8.3)	1 (11.1)	0 (0.0)	1 (6.7)	

Participants were stratified according to vaginal microbiota community clusters derived from shotgun metagenomic data. Continuous variables were compared across clusters using the Kruskal–Wallis test.

Categorical variables were compared using Fisher's exact test when expected cell counts were < 5 and the chi-square test otherwise, as appropriate. Statistically significant difference (*p* < 0.05) is indicated by ^*^.

To further characterize these patterns, we examined baseline characteristics by CST (Table 4). At the CST level, HPV type (*p* = 0.032), HPV vaccination status (*p* = 0.010), and hormone use (*p* = 0.019) differed significantly, whereas other demographic variables were broadly similar. Age showed a trend toward variation by CST (*p* = 0.054) but did not reach conventional statistical significance. In particular, vaccination status differed significantly across CSTs: unvaccinated women were overrepresented in CST IV, accounting for 60.0% of women in this CST, whereas vaccinated women were overrepresented in CST I, accounting for 90.9% of women in this CST. These findings suggest a potential association between vaccination and *Lactobacillus*-dominant community states. The significant difference in hormone use across CSTs indicates that hormonal exposure may additionally modulate CST distribution.

**Table 4:** Baseline characteristics of study participants by vaginal CST.

Variable	CST I (*n* = 11)	CST II (*n* = 1)	CST III (*n* = 16)	CST IV (*n* = 30)	CST V (*n* = 1)	*p*-value
Age (mean ± SD)	34.36 ± 7.07	39 ± NA	37.19 ± 4.31	39.2 ± 5.92	26 ± NA	0.054
Body weight, kg (mean ± SD)	57.25 ± 10.98	108.9 ± NA	59.88 ± 16.22	60.45 ± 14.2	39.6 ± NA	0.239
Educational attainment (*n*, %)						0.246
High school graduate	2 (18.2)	1 (100.0)	4 (25.0)	13 (43.3)	0 (0.0)	
College graduate	9 (81.8)	0 (0.0)	12 (75.0)	17 (56.7)	1 (100.0)	
Disease severity (*n*, %)						0.695
Pre-invasive	11 (100.0)	1 (100.0)	14 (87.5)	26 (86.7)	1 (100.0)	
Cervical cancer	0 (0.0)	0 (0.0)	2 (12.5)	4 (13.3)	0 (0.0)	
HPV infection (*n*, %)						0.492
No	0 (0.0)	0 (0.0)	1 (6.3)	0 (0.0)	0 (0.0)	
Yes	11 (100)	1 (100.0)	15 (93.8)	30 (100)	1 (100.0)	
HPV infection category (*n*, %)						0.510
No	0 (0.0)	0 (0.0)	1 (6.3)	0 (0.0)	0 (0.0)	
Single	5 (45.5)	1 (100.0)	10 (62.5)	15 (50.0)	1 (100.0)	
Multiple	6 (54.5)	0 (0.0)	5 (31.3)	15 (50.0)	0 (0.0)	
HPV vaccine status (*n*, %)						0.010^*^
No	1 (9.1)	1 (100.0)	6 (37.5)	18 (60.0)	0 (0.0)	
Yes	10 (90.9)	0 (0.0)	10 (62.5)	12 (40.0)	1 (100.0)	
HPV type (*n*, %)						0.032^*^
Negative	0 (0.0)	0 (0.0)	1 (6.3)	0 (0.0)	0 (0.0)	
Low risk	1 (9.1)	0 (0.0)	0 (0.0)	0 (0.0)	1 (100.0)	
16/18	2 (18.2)	0 (0.0)	8 (50.0)	14 (46.7)	0 (0.0)	
Other high risk	8 (72.7)	1 (100.0)	7 (43.8)	16 (53.3)	0 (0.0)	
Antibiotics (*n*, %)						1.000
No	11 (100.0)	1 (100.0)	15 (93.8)	28 (93.3)	1 (100.0)	
Yes	0 (0.0)	0 (0.0)	1 (6.3)	2 (6.7)	0 (0.0)	
Hormones (*n*, %)						0.019^*^
No	9 (81.8)	1 (100.0)	10 (62.5)	29 (96.7)	1 (100.0)	
Yes	2 (18.2)	0 (0.0)	6 (37.5)	1 (3.3)	0 (0.0)	
Probiotics (*n*, %)						0.371
No	7 (63.6)	0 (0.0)	5 (31.3)	12 (40.0)	0 (0.0)	
Yes	4 (36.4)	1 (100.0)	11 (69.8)	18 (60.0)	1 (100.0)	
Smoking (*n*, %)						0.813
No	9 (81.8)	1 (100.0)	9 (56.3)	21 (70.0)	1 (100.0)	
Yes	1 (9.1)	0 (0.0)	4 (25.0)	3 (10.0)	0 (0.0)	
Ex-smoker	1 (9.1)	0 (0.0)	3 (18.8)	6 (20.0)	0 (0.0)	
Drinking (*n*, %)						0.972
No	4 (36.4)	0 (0.0)	5 (31.3)	12 (40.0)	0 (0.0)	
Yes	7 (63.6)	1 (100.0)	11 (68.8)	18 (60.0)	1 (100.0)	
Diabetes (*n*, %)						0.492
No	11 (100.0)	1 (100.0)	15 (93.8)	30 (100.0)	1 (100.0)	
Yes	0 (0.0)	0 (0.0)	1 (6.3)	0 (0.0)	0 (0.0)	
Hypertension (*n*, %)						0.784
No	10 (90.9)	1 (100.0)	15 (93.8)	29 (96.7)	1 (100.0)	
Yes	1 (9.1)	0 (0.0)	1 (6.3)	1 (3.3)	0 (0.0)	

CSTs were defined based on dominant Lactobacillus species or diverse/non-Lactobacillus-dominant community structure inferred from shotgun metagenomic sequencing. CST I, L. crispatus-dominant; CST II, L. gasseri-dominant; CST III, L. iners-dominant; CST V, L. jensenii-dominant; CST IV, diverse/non-Lactobacillus-dominant microbiota.

Continuous variables were compared across CSTs using the Kruskal–Wallis test, and categorical variables using Fisher's exact test or the chi-square test, as appropriate. Statistically significant differences (*p* < 0.05) are indicated by ^*^.

### 3.4 Species-level microbiome composition

**Figure 3:**
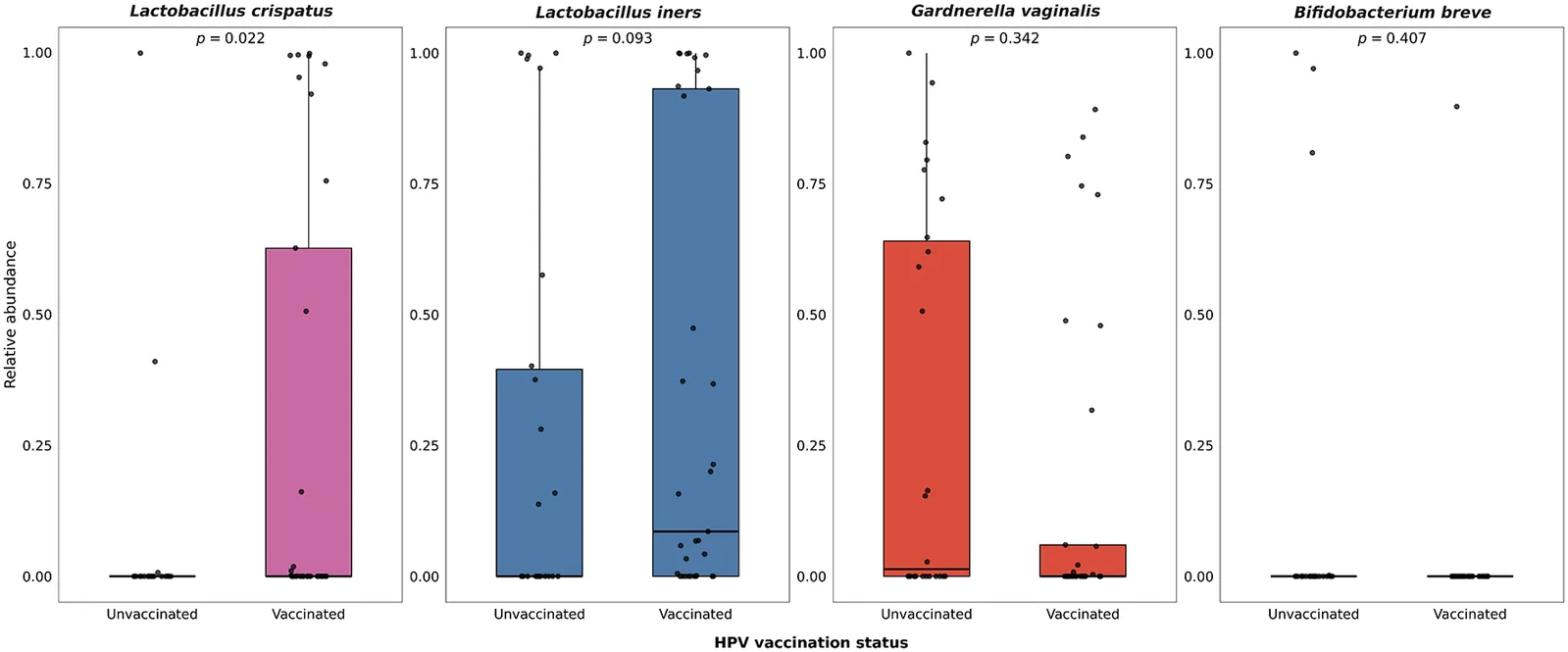
Species-level abundance of key taxa by HPV vaccination status. Box plots showing relative abundance of four major vaginal bacterial species (*L. crispatus, L. iners, G. vaginalis*, and *B. breve*) in HPV-unvaccinated and vaccinated women. Horizontal lines indicate medians; boxes the interquartile range (IQR); whiskers, 1.5 x IQR. *p*-values from Wilcoxon rank-sum tests comparing unvaccinated vs. vaccinated groups are displayed above each panel; *p* < 0.05 was considered statistically significant.

Given the cluster- and CST-level differences, we next examined the relative abundance of four key species by HPV vaccination status using Wilcoxon rank-sum tests (Figure 3). *L. crispatus* abundance was significantly higher (*p* = 0.022) in vaccinated women than in unvaccinated women, consistent with the pre-dominance of CST I in the vaccinated group. In contrast, although not significant, *G. vaginalis* appeared enriched in unvaccinated women, in line with the higher frequency of CST IV-like dysbiotic communities in this group. *L. iners* and *B. breve* showed more overlapping distributions between vaccinated and unvaccinated women, with no clear visual separation in relative abundance. These species-level patterns are consistent with the cluster-based results and suggest that HPV vaccination may be associated with higher *L. crispatus* abundance rather than broad shifts across *Lactobacillus* or other commensal taxa.

### 3.5 Multivariable analysis of the association between HPV vaccination and *L. crispatus*

To evaluate whether the observed association was independent of potential confounding factors, multivariable linear regression analysis was performed (Table 5). After adjustment for age, hormone use, antibiotics, probiotics, disease severity, HPV type, and multiple HPV infection, the positive association between HPV vaccination and *L. crispatus* abundance was attenuated (β = 0.205, 95% CI −0.083 to 0.493; *p* = 0.159). A sensitivity analysis excluding antibiotics and disease severity yielded similar results (β = 0.220, 95% CI −0.061 to 0.500; *p* = 0.122), indicating a consistent direction of association across models.

**Table 5:** Multivariable linear regression analysis of the association between HPV vaccination and *L. crispatus* abundance.

Variable	Full model β (SE)	Full model *p*-value	Sensitivity model β (SE)	Sensitivity model *p*-value
HPV vaccine (vaccinated vs. unvaccinated)	0.205 (95% CI: −0.083, 0.493)	0.159	0.220 (95% CI: −0.061, 0.500)	0.122
Age	−0.015 (0.012)	0.205	−0.014 (0.012)	0.229
Hormone use (yes vs. no)	0.058 (0.195)	0.767	0.039 (0.189)	0.838
Antibiotics (yes vs. no)	−0.210 (0.324)	0.520	—	—
Probiotics use (yes vs. no)	−0.193 (0.143)	0.184	−0.215 (0.136)	0.121
Disease severity (CC vs. pre-invasive)	−0.031 (0.253)	0.903	—	—
HPV type (16/18 vs. other)	−0.203 (0.162)	0.217	−0.200 (0.143)	0.166
Multiple HPV (yes vs. no)	0.025 (0.148)	0.865	0.039 (0.143)	0.786
Metric	Full model	Sensitivity model		
*N*	59	59		
*R* ^2^	0.188	0.180		
Adjusted *R*^2^	0.058	0.086		
*F*-statistic (df)	1.448 (8, 50)	1.906 (6, 52)		
Overall model *p*-value	0.203	0.097		
Residual standard error	0.508	0.501		

Results are shown for the full model adjusted for age, hormone use, antibiotics, probiotics, disease severity, HPV type, and multiple HPV infection, and for a sensitivity model excluding antibiotics and disease severity.

Model fit statistics (N, R^2^, F-statistic, overall p-value) are shown at the bottom of the table.

β, regression coefficient; SE, standard error; CI, confidence interval.

## 4 Discussion

The VM is a major determinant of female overall reproductive health. A “healthy” VM is typically characterized by dominance of *Lactobacillus* species, particularly *L. crispatus*, with smaller contributions from *L. gasseri* and *L. iners*. In contrast, high-diversity communities enriched for anaerobes are associated with genital tract inflammation and adverse outcomes (Norenhag et al., 2020; Wei et al., 2022; Yang et al., 2025). The role of *L. iners* remains debated, as it is frequently detected in apparently healthy women but has also been linked to transitional or dysbiotic states (Lee et al., 2013; Munoz et al., 2021; Sun et al., 2021; Colbert et al., 2023). Multiple studies have reported a bidirectional relationship between HPV infection and the VM: compared with HPV-negative women, HPV-positive women commonly show higher microbial diversity and a reduced abundance of protective *Lactobacillus* spp. However, not all HPV infections including those with high-risk genotypes are associated with marked microbiota disruption, suggesting that the impact of HPV on the VM may depend on infection persistence and the downstream pathological outcome (Lee et al., 2013; Sun et al., 2021; Lebeau et al., 2022; Zhang et al., 2022; Jung et al., 2025).

Despite widespread implementation of prophylactic HPV vaccination—which is highly effective against vaccine-type hrHPV infection and related high-grade lesions—hrHPV infections persist in a subset of women and can progress to cervical lesions. In our cohort, genotype-level data indicate that many persistent infections in vaccinated women involved non-vaccine hrHPV types or genotypes not covered by the particular vaccine received, rather than failure of the vaccine against its targeted types. While numerous studies have evaluated associations between HPV infection and VM composition, far less is known about how HPV vaccination status relates to the VM among women with established cervical lesions and persistent hrHPV infection. To address this gap, we conducted a cross-sectional analysis profiling the VM of women with histologically confirmed cervical lesions, stratified by HPV vaccination status. Using metagenomic shotgun sequencing, we characterized microbial communities at high taxonomic resolution. These findings suggest that VM composition differs between unvaccinated and vaccinated women, consistent with a potential association between HPV vaccination and microbiota structure.

At the community level, alpha diversity did not differ significantly between vaccinated and unvaccinated women, indicating comparable within-sample microbial diversity despite differences in community composition. Using Bray-Curtis dissimilarities, PERMANOVA suggested that vaccination status was associated with overall VM composition, and PERMDISP indicated that this difference was unlikely to be driven by unequal dispersion between groups. In stacked bar plots, vaccinated women more frequently showed communities dominated by *L. crispatus* and *L. iners*, whereas unvaccinated women more often harbored higher-diversity communities enriched for taxa such as *G. vaginalis* and *B. breve*. These findings suggest that vaccination status is related to overall community configuration rather than to large shifts in diversity metrics alone.

To examine community structure in more detail, we classified samples into eight clusters and five CSTs based on species composition and relative abundance. Consistent with previous reports in Korean and other East Asian cohorts, CST II and CST V were rare and showed no clear association with vaccination status, consistent with their generally low prevalence in this population (Lee et al., 2013; Munoz et al., 2021; Colbert et al., 2023). In contrast, CST I was significantly more prevalent among vaccinated women, whereas CST IV was more frequent among unvaccinated women. CST III appeared numerically more frequent in vaccinated women, although this difference did not reach statistical significance.

These patterns align with longitudinal data indicating that *Lactobacillus*-dominant CSTs are associated with higher hrHPV clearance rates, whereas CST IV communities enriched for *Gardnerella, Atopobium, Sneathia, Megasphaera*, and *Prevotella* predict hrHPV persistence and progression to CIN2+ lesions (Dareng et al., 2020; Zeng et al., 2023; Bautista et al., 2025). In contrast, *L. iners*–dominant CST III is regarded as an intermediate, relatively unstable state that can transition toward either *L. crispatus*–dominated eubiosis or high-diversity dysbiosis, and is observed both in women who clear hrHPV and in those with recurrent dysbiosis, highlighting its context-dependent role (Di Pierro et al., 2021; Zheng et al., 2021; Liu et al., 2024; Pai et al., 2025). In this context, the higher prevalence of CST I and numerical increase in CST III among vaccinated women in our cohort are consistent with the established protective association of *L. crispatus* and the potential transitional role of *L. iners*. These observations generate the hypothesis that HPV vaccination and an *L. crispatus-*dominant VM may jointly contribute to favorable HPV-related outcomes, although this possibility requires evaluation in longitudinal studies.

At the species level, we focused on four key taxa that characterized the main CSTs and clusters: *L. crispatus, L. iners, G. vaginalis*, and *B. breve*. Using Wilcoxon rank-sum tests, vaccinated women showed higher relative abundance of *L. crispatus* than unvaccinated women, whereas *G. vaginalis* tended to be enriched in unvaccinated women without reaching conventional statistical significance. *L. iners* and *B. breve* displayed considerable overlap between groups, consistent with their presence across CSTs and clusters. These species-level patterns mirror the CST and cluster results, suggesting that HPV vaccination may be associated with increased *L. crispatus* abundance and lower representation of *G. vaginalis*–enriched, CST IV-like communities, rather than with broad changes across all *Lactobacillus* or commensal taxa.

Within a broader ecological framework, observational studies have shown that multiple host and environmental factors (e.g., reproductive-age hormonal milieu, combined hormonal contraception, local estrogen therapy, and *Lactobacillus*-containing probiotics) favor *Lactobacillus*-dominated vaginal communities, whereas age-related estrogen decline, dysbiosis-inducing antibiotic regimens, and hrHPV infection are associated with higher-diversity, non-*Lactobacillus* CSTs (Mayer et al., 2015; Mitra et al., 2020; Nieto et al., 2025; Udjianto et al., 2025). Our findings extend this framework by suggesting that prophylactic HPV vaccination status may represent an additional characteristic associated with *L. crispatus*-dominant communities in reproductive-age women with cervical lesions. However, as vaccination was not randomly assigned, this association should be interpreted cautiously, as discussed later in this section.

Age, estrogen status, hormonal contraception, systemic antibiotics, and probiotic use are all well-established determinants of vaginal community structure and stability. Reproductive-age women with sufficient estrogen typically exhibit *Lactobacillus*-dominated communities, whereas peri- and post-menopausal women more frequently show higher-diversity, BV-like microbiota (Muhleisen and Herbst-Kralovetz, 2016; Moore et al., 2024), which informed our exclusion criteria (post-menopausal status or age ≥50 years) to avoid these well-characterized confounders. Within this range, we nonetheless observed that hormone use differed by CST and appeared to influence *L. iners-*dominated communities, consistent with previous reports that hormonal exposures can stabilize or destabilize specific CSTs (Thomas-White et al., 2020; Kuciel et al., 2025). Standard antibiotic regimens for BV (e.g., oral metronidazole) often induce a transient shift from CST IV to *Lactobacillus* dominance, but recurrences are common. In contrast, *Lactobacillus*-based probiotics administered orally or intravaginally repeatedly demonstrate the ability to convert CST IV to *Lactobacillus*-dominant communities and reduce BV recurrence (Liu P. et al., 2023; Udjianto et al., 2025). Against this complex background of determinants, our data raise the possibility that HPV vaccination is associated with *L. crispatus*-dominant microbiota in some women with cervical lesions. Any potential links with mitigation of hrHPV-associated inflammation or epithelial disruption remain speculative and were not directly tested in this study.

Taken together, our community-, cluster-, and species-level analyses suggest that HPV-vaccinated women with cervical lesions are more likely than unvaccinated women to harbor *L. crispatus–*dominant CST I and higher *L. crispatus* abundance, even though overall alpha diversity and most demographic and clinical variables were similar between groups. These observations align with prior longitudinal work linking *Lactobacillus*-dominated communities to more hrHPV clearance and persistence outcomes (Brotman et al., 2014; Mitra et al., 2015; Bautista et al., 2025), and support the hypothesis that HPV vaccination status is jointly associated with a more favorable vaginal ecological niche, although our cross-sectional design and the high hrHPV positivity observed even among vaccinated women preclude any inference about actual hrHPV clearance in this cohort.

The high hrHPV positivity among vaccinated women should be interpreted in the context of this clinically selected cohort. All participants had histologically confirmed cervical lesions; therefore, vaccinated women with breakthrough or persistent HPV infection were inherently overrepresented, and the observed prevalence cannot be interpreted as vaccine failure or as an estimate of vaccine effectiveness in the general population. In addition, vaccination occurred relatively late in this cohort (mean age, 29.3 years), raising the possibility of HPV exposure before vaccination. Because HPV vaccines are prophylactic and do not clear pre-existing infection or treat established lesions, prior infection, together with the detection of non-vaccine hrHPV genotypes, may explain the high hrHPV positivity observed among vaccinated participants.

Although HPV vaccination temporally precedes vaginal sampling, our cross-sectional design limits definitive causal inference. Because vaccination was not randomly assigned, women who received HPV vaccination may differ systematically from unvaccinated women in birth cohort, socioeconomic status, health awareness, healthcare-seeking behavior, screening frequency, sexual behavior, contraceptive use, or previous gynecologic care, several of which may also influence VM composition. Vaccination status may therefore partly represent these unmeasured demographic, behavioral, or healthcare-related characteristics rather than a direct biological effect of vaccination. Although age and several measured clinical factors were included in the analyses, residual and unmeasured confounding cannot be excluded. Additionally, we lacked granular data on vaccination timing relative to HPV exposure and on completion of the full recommended dose schedule (number of doses), as well as detailed hormonal contraception or menopausal hormone therapy specifics, systemic antibiotic exposures beyond the washout period, or probiotic use. Given that current HPV vaccines are prophylactic and do not clear pre-existing infections, this limits our ability to distinguish the contributions of prior infection, partial vs. complete vaccination, non-vaccination hrHPV types to the high hrHPV positivity observed in vaccinated women. Each of these represents potential confounders or mediators of *Lactobacillus*-dominance status. Residual confounding by sexual behavior patterns and partner-level factors also cannot be excluded. Finally, our small sample size (*n* = 59), exclusion of post-menopausal women, and lack of longitudinal cancer-specific clinical outcomes further limits generalizability and clinical relevance across the female lifespan and disease contexts.

We recommend future observation longitudinal cohorts that enroll women prior to HPV vaccination and follow them with repeated cervicovaginal sampling while capturing all established microbiota determinants (age, menstrual status, contraceptive/hormone replacement therapy (HRT), antibiotics, probiotics). Additionally, further multi-omics validation is needed to confirm functional shifts in vaccination-associated *L. crispatus*-dominant communities (e.g., lactate/H_2_O_2_/bacteriocin pathways, HPV-driven inflammation), alongside prospective tracking of cancer outcomes (lesion regression, recurrence risk, survival) to establish whether vaccine-associated *L. crispatus* enrichment translates to improved cervical health and prognosis.

## 5 Conclusion

HPV vaccination was associated with higher prevalence of *L. crispatus*-dominant communities (CST I) and lower prevalence of diverse communities (CST IV) in our cohort of women with cervical lesions. Given the cross-sectional design, these findings reflect an association between vaccination status and vaginal community state type, rather than a causal or temporal relationship.

## Data Availability

The datasets presented in this study can be found in online repositories. The names of the repository/repositories and accession number(s) can be found below: https://www.ebi.ac.uk/ena, PRJEB122097.
