# Supplementary material for: HPV vaccination associates with Lactobacillus crispatus-dominant vaginal microbiota in women with cervical lesions: a metagenomic study
Source: Front Microbiol. 2026 Aug 14;17:1906211. doi: 10.3389/fmicb.2026.1906211 (PMC13522137; doi:10.3389/fmicb.2026.1906211)
Supplement: Supplementary file 1 [file Table_1.docx]

Supplementary Material

# 1 Supplementary Figures

**Total Cohort (n = 98)**

Women with confirmed cervical lesions

**Excluded (n = 39)**

Postmenopausal (≥ 50 years)

**Included (n = 59)**

- Cervical lesion confirmed
- Pre-menopausal (< 50 years)

**Supplementary Figure 1.** **Overall cohort schema.** A total of 98 women with confirmed cervical lesions were screened, and 59 premenopausal women (< 50 years) were included after excluding 39 postmenopausal participants (≥ 50 years).

**Supplementary Figure 2. Elbow plots supporting an optimal cluster number of k = 8 for vaginal microbiota clustering.** Within-cluster sum of squares (WSS) across candidate numbers of clusters (k = 1–20) for vaginal microbiota profiles at the (A) genus and (B) species levels. In both cases, the elbow of WSS curve support k = 8 as the optimal number of clusters for subsequent community clustering based on shotgun metagenomic data from 59 vaginal samples.
